# The effect of auricular vagus nerve stimulation in women with temporomandibular joint disorders: a randomized controlled study

**DOI:** 10.1590/1806-9282.20241739

**Published:** 2025-06-02

**Authors:** Alper Percin, Hande Basat, Ali Veysel Ozden, Semiha Yenisehir

**Affiliations:** 1 Avrasya University, Faculty of Health Sciences, Department of Physiotherapy and Rehabilitation – Trabzon, Türkiye.; 2 Bahçeşehir University, Faculty of Health Sciences, Department of Physiotherapy and Rehabilitation – İstanbul, Türkiye.; 3 Ordu University, Faculty of Health Sciences, Department of Physiotherapy and Rehabilitation – Ordu, Türkiye.

**Keywords:** Autonomic nervous systems, Myofascial trigger point pain, Pain management, Temporomandibular disorders, Vagus nerve stimulation

## Abstract

**OBJECTIVE:**

Temporomandibular disorders associated with myofascial pain syndrome cause pain and disability in daily life. The aim of this study was to investigate the effect of auricular vagus nerve stimulation on pain in women with myofascial pain syndrome-related temporomandibular disorder.

**METHODS:**

A total of 50 women with myofascial pain syndrome-related temporomandibular disorder aged between 18 and 35 years participated in this study. The vagus group (n=25) received auricular vagus nerve stimulation and manual therapy and exercise, and the control group (n=25) received only manual therapy and exercise twice a week for 3 months. The pressure pain threshold was used for the assessment.

**RESULTS:**

In the vagus group, pressure pain threshold on the masseter, temporalis, sternocleidomastoid, digastricus, trapezius, and levator scapula muscles increased statistically significantly after treatment compared to baseline (p<0.05). In the control group, pressure pain threshold on the masseter and levator scapula muscles increased (p<0.05), while no statistically significant difference was found in the pressure pain threshold on the temporalis, sternocleidomastoid, digastricus, and trapezius muscles (p>0.05). When both groups were compared, vagus group was found to be more effective than control group in increasing pressure pain threshold in the masseter, trapezius, and levator scapula muscles (p<0.05).

**CONCLUSION:**

Auricular vagus nerve stimulation was found to be effective in increasing pressure pain threshold in patients with myofascial pain syndrome-related temporomandibular disorder. The clinical trial registration number was NCT05500716.

## INTRODUCTION

Temporomandibular disorders (TMDs) are a group of disorders that affect the muscles used to chew, the temporomandibular joint (TMJ), and related structures^1^. The symptoms of TMDs include decreased or excessive range of motion (ROM), clicking or crepitation in the joint, pain around the joint or muscle group, problems with chewing and swallowing, headaches, bruxism, and neck pain^2^. According to the Diagnostic Criteria for Temporomandibular Disorders (DC/TMDs) classification system, TMDs are divided into four main subtypes: myalgia, arthralgia, disc displacement, and degenerative disc disease^3^. Myalgia is muscle pain which localizes in the jaw and affects TMJ function and has two subtypes, such as local myalgia and myofascial pain. Myofascial pain syndrome (MPS) is characterized by clenching and grinding of the teeth, repetitive trauma, and overuse of the masticatory muscles, resulting in pain, trigger points, and spasms^4^.

The development of sympathetic hyperactivity as a result of autonomic nervous system (ANS) dysfunction is known to be a factor in the severity of TMD-related symptoms^5^. TMDs may reflect changes in sympathoadrenal and inflammatory cytokine function in response to stressors, and increased sympathetic activity may lead to long-term disability^6^. TMD is a condition that occurs in the presence of ANS dysfunction and elevated stress levels and can be characterized by widespread pain symptoms^7^.

Auricular vagus nerve stimulation (aVNS) is a neuromodulation technique with disease-modifying effects and sustainable therapeutic interventions ranging from chronic pain, neurodegenerative and metabolic disorders, to inflammatory and cardiovascular diseases^8^. VNS has a clinical significance in decreasing pain by non-pharmacological pathways^9^. The mechanisms such as inhibition of pro-inflammatory factors may be involved in the treatment of pain with VNS by suppressing inflammation. The aVNS procedure stimulates the dorsal motor nucleus of the vagus nerve in the brainstem. From this nucleus, neurons extend along the vagus nerve to the superior mesenteric ganglion complex, promoting the release of norepinephrine in the splenic parenchyma and triggering the release of acetylcholine from T-cells. Acetylcholine inhibits the release of tumor necrosis factor and pro-inflammatory cytokines in macrophages^10^. In this way, pain relief is achieved by suppressing inflammation^11^.

In this study, it was hypothesized that aVNS could be effective in decreasing pain in the treatment of MPS-related TMD. The aim of this study was to investigate the effects of aVNS as an adjunct to an intervention method on pain relief in patients with MPS-related TMD.

## METHODS

### Design and sampling

This prospective, single-center, assessor-blind, randomized controlled trial was conducted at Igdır Oral and Dental Health Centre between October 2022 and March 2023 according to the guidelines of the Declaration of Helsinki. All participants signed the informed consent form. Ethical approval was obtained from the Non-Invasive Clinical Research Ethics Committee of the Medical Faculty of Igdır University (protocol code: 80576354-050-99/103 and approval date: 26/10/2022). The clinical trial registration number was NCT05500716. A written informed consent was obtained from each patient.

A total of 50 women diagnosed with MPS-related TMD according to DC/TMD classification were included in this study. As the prevalence of MPS-related TMD pain is increasing in females and there is a growing gender gap, it was planned to include female patients in this study. Inclusion criteria were determined as being between the ages of 18 and 35 years and diagnosed with MPS-related TMD according to the DC/TMD classification. Exclusion criteria were having a history of a surgical or invasive procedure on the TMJ, having neurological or psychiatric diseases, being pregnant, presence of infection or a tumor structure in intraoral structures, and having undergone a surgical procedure in the cervical region. Informed consent was obtained from the study participants.

The study participants (n=50) were randomly divided into two groups: vagus group (VG) (n=25) and control group (CG) (n=25). The participants were randomly assigned to the two groups separately and in equal numbers using software (https://www.randomizer.org). Each patient was asked to choose a random number between 1 and 50. Depending on the group to which the participants belonged, treatment planning was then carried out. Assessments were made by another researcher (blinded assessor) at baseline and after the intervention period.

### Instruments

#### Pressure pain threshold

Pressure pain threshold (PPT) was measured using the Baseline^®^ Dolorimeter device (30 kg×1/4 kg). Painful trigger points were located by palpation on the masseter, temporalis, sternocleidomastoid (SCM), digastric, upper trapezius, and levator scapula muscles, and the PPT measurement was recorded.

### Interventions

Participants attended this study for a period of 3 months, two sessions per week, divided into CG and VG. The VG received aVNS and manual therapy and exercise (MT-E), while the CG received only MT-E.

#### Deep friction massage

Deep friction massage (DFM) was performed with the patients in a comfortable position, the masseter and temporalis muscles located by palpation, and DFM initiated. DFM was completed in an average of 5 min.

#### Rocabado exercises

The Rocabado exercise program includes tongue relaxation technique, TMJ rotation control, rhythmic stabilization technique, neck axial extension, shoulder girdle posture, and stabilized head flexion. The participants were trained to perform the exercises six times a day with six repetitions of each movement.

#### Vagus nerve stimulation

**Figure 1:**
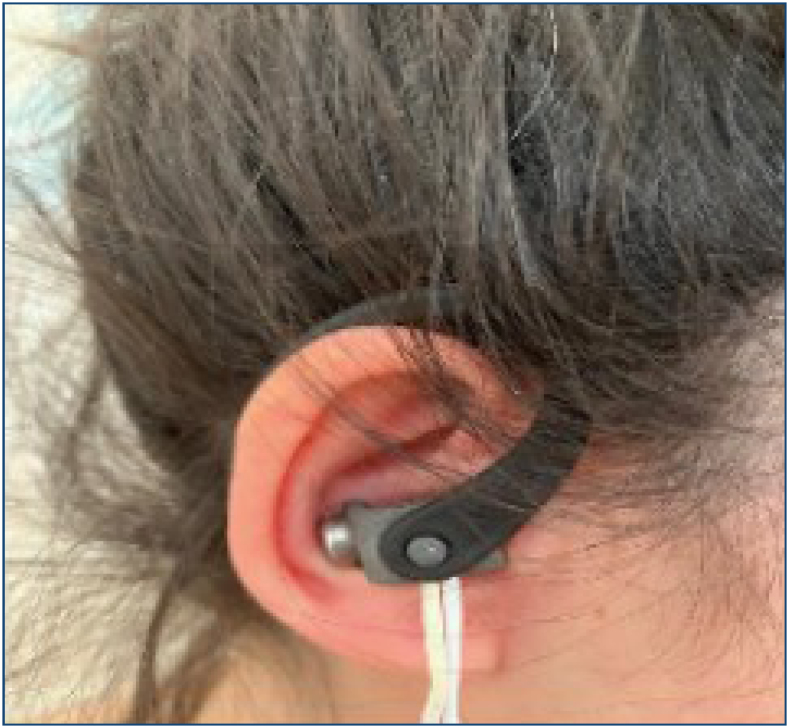
Vagus nerve stimulation and the Vagustim device.

The Vagustim device (Copyright Vagustim, 2023, Vagustim Health Technologies, San Francisco, CA, USA) was used for vagal neuromodulation in this study (Figure 1). The aVNS protocol consisted of bilateral auricular stimulation, with a duration of 20 min, 10 Hz stimulation frequency, 250 μs pulse width, suprathreshold current (0.13–50 mA), and biphasic mode, inspired by the summary range of parameters from a previous study^12^. The current intensity was increased to the level tolerated by the patient. Stimulation was applied from the tragus and concha, where the fibers of the auricular vagus nerve are mainly located. aVNS was only used on rehabilitation days, before MT-E.

### Data analysis

The sample size was calculated with G*power 3.1.9.6 software (MAC version). Effect size=0.25, alpha level=0.05, and beta level=0.15 (85% power ratio) were selected, and finally, 56 participants comprised the total sample size. The SPSS 28.0.1 (MAC version, Chicago, USA) was used for statistical analysis. The Kolmogorov-Smirnov and Shapiro-Wilk tests were used to assess the normality of the values obtained from the participants. When comparing dependent groups, a paired-sample t-test was used when the parametric test assumption was met, and the Wilcoxon paired two-sample test was used when the parametric test assumption was not met. The level of statistical significance was accepted as p<0.05.

## RESULTS

The physical characteristics are shown in Table 1. The mean age of all the participants was 20.5 years, height was 163.5 cm, weight was 56.5 kg, and body mass index was 21 (kg/m^2^). The physical characteristics of the groups were similar (p>0.05).

**Table 1:** Physical characteristics of the groups.

Variables	CG (n=25)	VG (n=25)	p	Skewness/kurtosis
	Mean±SD	Mean±SD		
Age (years)	20±1.36	21±4.19	0.543	0.512–0.992
Height (cm)	163±6.20	164±5.26	0.200	0.512–0.992
Weight (kg)	56±7.52	57±6.50	0.070	0.512–0.992
BMI (kg/m^2^)	22±3.71	20±6.64	0.088	0.512–0.992

CG: control group; VG: vagus group; BMI: body mass index; SD: standard deviation; n: number of participants; p>0.05; cm: centimeters; kg: kilograms.

There was no statistical difference between the groups at the baseline according to PPT (p>0.05) (Table 2). After treatment, there was a significant increase in the PPT in the masseter, trapezius, and levator scapula muscles in the VG compared to the CG (p<0.05) (Table 2).

**Table 2:** Comparison of pressure pain threshold of the groups at baseline and after treatment.

Baseline variables	CG (mean±SD)	VG (mean±SD)	t	p
**PPT**			**t**	
Masseter	0.4±0.29	0.5±0.32	0.395	0.306
			**U**	
Temporalis	0.8±0.56	0.8±0.71	0.323	0.305
SCM	0.7±0.62	0.7±0.63	0.489	0.121
			**t**	
Digastricus	0.4±0.25	0.6±0.34	0.987	0.324
Trapezius	2.2±0.85	2.2±0.85	0.665	0.829
Lev. scapula	3.3±0.83	3.1±3.74	0.512	0.898
After-treatment variables	CG (mean±SD)	VG (mean±SD)	t	p
**PPT**			**t**	
Masseter	0.6±0.44	0.9±0.84	2.111	0.003*
			**U**	
Temporalis	0.9±0.57	0.9±0.94	0.158	0.419
			**t**	
SCM	1.1±0.94	1.4±0.99	0.392	0.833
Digastricus	0.6±0.47	0.8±0.59	0.460	0.861
Trapezius	2.4±0.71	3.1±1.30	3.335	0.033*
Lev. scapula	3.7±0.83	3.7±0.83	2.161	0.047*

CG: control group; VG: vagus group; PPT: pressure pain threshold; SD: standard deviation; SCM: sternocleidomastoid; t: independent-sample t-test; U: Mann-Whitney U test;

* p<0.05.

The comparison of intra-group PPT is shown in Table 3. There was a statistically significant difference in the muscles of masseter and levator scapula in the CG after treatment compared to baseline (p<0.05). There was a statistically significant difference in the muscles of masseter, trapezius, SCM, digastricus, trapezius, and levator scapula in the VG after the intervention compared to baseline (p<0.05).

**Table 3:** Intra-group comparisons of pressure pain threshold.

CG	Baseline variables (kg/cm^2^) mean±SD	AT variables (kg/cm^2^) mean±SD	T	p
Masseter	0.43±0.29	0.61±0.44	0.603	0.028*
			**Z**	**p**
Temporalis	0.83±0.56	0.86±0.57	0.176	0.441
			**t**	**p**
SCM	0.65±0.62	1.03±0.94	0.475	0.470
Digastricus	0.43±0.25	0.64±0.47	0.523	0.304
Trapezius	2.15±0.85	2.35±0.71	0.307	0.186
Lev. scapula	3.28±0.83	3.73±0.83	0.944	0.002*
VG	Baseline variables (kg/cm^2^) (mean±SD)	AT variables (kg/cm^2^) (mean±SD)	t	p
Masseter	0.48±0.32	0.93±0.84	1.466	0.014*
			**Z**	**p**
Temporalis	0.77±0.71	0.98±0.94	0.158	0.047*
			**t**	**p**
SCM	0.74±0.63	1.41±0.99	0.392	0.004*
			**Z**	**p**
Digastricus	0.64±0.34	0.88±0.59	0.460	0.010*
			**t**	**p**
Trapezius	2.19±0.85	3.13±1.30	738	0.006*
Lev. scapula	3.12±3.74	3.74±0.83	1.006	0.001*

CG: control group; VG: vagus group; n: number of participants; SD: standard deviation; AT: after treatment; SCM: sternocleidomastoid; t: paired-sample t-test; Z: Wilcoxon test; p>0.05,

* p<0.05.

## DISCUSSION

This study investigated the effects of aVNS on PPT in female patients with MPS-related TMD. After treatment, aVNS with MT-E was found to be more effective in improving PPT compared to the only-MT-E program.

Pain is one of the most common symptoms reported by patients with MPS-related TMD. Tapiainen et al. investigated the effects of tooth clenching and continuous activation of the masticatory muscles on the ANS, using a protocol of two 5-s maximal clenches followed by a 5-s interval and then a maximal prolonged clench until the participant felt pain. They concluded that clenching of the teeth causes sympathetic hyperactivity to predominate and consequently can cause pain^13^. Galhardo et al. investigated the TMD-Pain Screener Questionnaire and the Depression, Anxiety and Stress Scale (DASS-21) to detect the presence of social isolation-induced TMDs and pain in medical students during the pandemic, considering that negative emotional changes may cause TMDs and related pain^14^. The results of the study showed that TMDs and pain had a strong association with high depression, anxiety, and stress scores in students. In the present study, the participants had a lower PPT and complained of pain at baseline. After aVNS intervention, an increase in PPT was observed.

The use of aVNS in chronic pain conditions and its successful outcomes are documented in the literature. Kovacic et al. achieved a significant reduction in abdominal pain symptoms with aVNS in adolescents with gastrointestinal disorders^15^. Najib et al. studied 12 weeks of aVNS treatment in patients diagnosed with migraine and concluded that aVNS was effective in preventing migraine pain and attacks^16^. Meints et al. investigated the effects of aVNS with cognitive meditation on chronic low back pain and found that aVNS was an effective method for pain relief^17^. Aranow et al. investigated the effects of aVNS on 18 patients diagnosed with systemic lupus erythematosus and experiencing symptoms of pain and fatigue. It was found that pain and fatigue scores decreased after aVNS^18^. Sator-Katzenschlager et al. found that the results of aVNS for acute pain were less effective than the results for chronic pain^19^. In another study, Zheng et al. investigated the relationship between age, gender, pain, and PPT in individuals with TMDs and found that there was a correlation between age, gender, and PPT, but there was no correlation between subjective pain and PPT^20^.

PPT is defined as the minimum force required to produce a painful sensation, and a decrease in PPT is common in MPS-related TMD. Takeuchi et al. investigated the administration of a low-level clenching task, which could induce TMD symptoms. After performing the task, a significant decrease in PPT was found in the temporalis and masseter muscles^21^. Greenspan et al. investigated PPT and ANS-related factors in the development of TMDs. PPT in the masseter and temporalis muscles and heart rate variability were measured in the ANS assessment^22^. They found that an increase in heart rate was associated with increased sympathetic activity and a decrease in PPT in the masseter and temporalis muscles in patients with TMDs. Ünal et al. investigated the effects of aVNS on MPS and found an increase in trapezius muscle PPT with aVNS compared to the CG^23^. In the present study, an increase in the PPT of the masseter, temporalis, and levator scapula muscles was observed after aVNS and MT-E compared to MT-E-only program. This suggests that aVNS may be an effective method for increasing PPT.

Galhardo et al. investigated the relationship between menopausal symptoms and TMD-related pain. According to the criteria of the Reproductive Ageing Workshop, participants in the late menopausal transition, early postmenopausal, and late postmenopausal periods were included, and their TMD-induced pain was assessed^24^. The results of the study showed that TMD-related pain and menopausal symptoms were related and that pain was more severe in the late menopausal transition period. Galhardo et al., based on the hypothesis that sex hormones are effective in the development of TMDs, investigated the receptor expression of age and sex factors in the masticatory muscles of rats^25^. The results of the study showed that estrogen receptor expression was found to be low in all muscles and groups, suggesting the effect of sex hormones on TMDs.

This study has some limitations. First, sham stimulation with MT-E or active stimulation without MT-E method would have shown better results. Second, the small number of participants is another limitation of this study. The strengths of this study are that MT-E (active treatment method) was performed in the CG and some improvements were observed.

In conclusion, the fact that the disease progresses with different symptoms in TMDs makes the treatment of TMDs difficult. aVNS aims to maintain the balance between sympathetic activity and parasympathetic activity by stimulating vagus fibers, which explains its therapeutic effect. aVNS can improve the quality of life in patients with TMDs, primarily by decreasing pain. aVNS has shown positive results in the treatment of MPS-related TMD in terms of increasing the PPT and decreasing pain. In the future, long-term effects should be investigated with different stimulation parameters and methods. In light of these results, there is a need for more detailed assessment methods and larger populations regarding the physiological effects of aVNS in the treatment of MPS-related TMD.
